# Platelet-derived circulating soluble P-selectin is sufficient to induce hematopoietic stem cell mobilization

**DOI:** 10.1186/s13287-023-03527-w

**Published:** 2023-10-20

**Authors:** Tso-Fu Wang, Yu-Shan Liou, Shang-Hsien Yang, Guan-Ling Lin, Ya-Wen Chiang, Te-Sheng Lien, Chi-Cheng Li, Jen-Hung Wang, Hsin-Hou Chang, Der-Shan Sun

**Affiliations:** 1 Department of Hematology and Oncology, Hualien Tzu Chi Hospital, Buddhist Tzu Chi Medical Foundation, Hualien, Taiwan, Republic of China; 2 Department of Medicine, College of Medicine, Tzu Chi University, Hualien, Taiwan, Republic of China; 3 Buddhist Tzu Chi Stem Cells Center, Hualien Tzu Chi Hospital, Buddhist Tzu Chi Medical Foundation, Hualien, Taiwan, Republic of China; 4 Department of Molecular Biology and Human Genetics, College of Medicine, Tzu Chi University, No. 701, Section 3, Zhong-Yang Road, Hualien, 97004 Taiwan, Republic of China; 5 Department of Pediatric Hematology and Oncology, Hualien Tzu Chi Hospital, Buddhist Tzu Chi Medical Foundation, Hualien, Taiwan, Republic of China; 6 Present Address: Integration Center of Traditional Chinese and Modern Medicine, Hualien Tzu Chi Hospital, Buddhist Tzu Chi Medical Foundation, Hualien, Taiwan, Republic of China; 7 Center of Stem Cell and Precision Medicine, Hualien Tzu Chi Hospital, Buddhist Tzu Chi Medical Foundation, Hualien, Taiwan, Republic of China; 8 Department of Medical Research, Hualien Tzu Chi Hospital, Buddhist Tzu Chi Medical Foundation, Hualien, Taiwan, Republic of China

**Keywords:** Granulocyte colony-stimulating factor, Hematopoietic stem cell mobilization, Soluble P-selectin

## Abstract

**Background:**

Granulocyte colony-stimulating factor (G-CSF)-mediated mobilization of hematopoietic stem cells (HSCs) is a well-established method to prepare HSCs for transplantation nowadays. A sufficient number of HSCs is critical for successful HSC transplantation. However, approximately 2–6% of healthy stem cell donors are G-CSF-poor mobilizers for unknown reasons; thus increasing the uncertainties of HSC transplantation. The mechanism underlining G-CSF-mediated HSC mobilization remains elusive, so detailed mechanisms and an enhanced HSC mobilization strategy are urgently needed. Evidence suggests that P-selectin and P-selectin glycoprotein ligand-1 (PSGL-1) are one of the cell–cell adhesion ligand–receptor pairs for HSCs to keep contacting bone marrow (BM) stromal cells before being mobilized into circulation. This study hypothesized that blockage of PSGL-1 and P-selectin may disrupt HSC-stromal cell interaction and facilitate HSC mobilization.

**Methods:**

The plasma levels of soluble P-selectin (sP-sel) before and after G-CSF administration in humans and male C57BL/6J mice were analyzed using enzyme-linked immunosorbent assay. Male mice with P-selectin deficiency (*Selp*^−/−^) were further employed to investigate whether P-selectin is essential for G-CSF-induced HSC mobilization and determine which cell lineage is sP-sel derived from. Finally, wild-type mice were injected with either G-CSF or recombinant sP-sel to investigate whether sP-sel alone is sufficient for inducing HSC mobilization and whether it accomplishes this by binding to HSCs and disrupting their interaction with stromal cells in the BM.

**Results:**

A significant increase in plasma sP-sel levels was observed in humans and mice following G-CSF administration. Treatments of G-CSF induced a decrease in the level of HSC mobilization in *Selp*^−/−^ mice compared with the wild-type (*Selp*^+/+^) controls. Additionally, the transfer of platelets derived from wild-type mice can ameliorate the defected HSC mobilization in the *Selp*^−/−^ recipients. G-CSF induces the release of sP-sel from platelets, which is sufficient to mobilize BM HSCs into the circulation of mice by disrupting the PSGL-1 and P-selectin interaction between HSCs and stromal cells. These results collectively suggested that P-selectin is a critical factor for G-CSF-induced HSC mobilization.

**Conclusions:**

sP-sel was identified as a novel endogenous HSC-mobilizing agent. sP-sel injections achieved a relatively faster and more convenient regimen to mobilize HSCs in mice than G-CSF. These findings may serve as a reference for developing and optimizing human HSC mobilization in the future.

**Supplementary Information:**

The online version contains supplementary material available at 10.1186/s13287-023-03527-w.

## Background

Hematopoietic stem cells (HSCs) exert the ability to self-renew and differentiate into all kinds of hematopoietic cells (red blood cells, white blood cells, and platelets) in human body [1]. HSC transplantation has been used to treat hematological diseases for over 50 years [2, 3]. Transplantation with granulocyte colony-stimulating factor (G-CSF)-mobilized HSCs is more convenient and popular than transplantation using bone marrow (BM) HSCs because of lower cost, faster engraftment, and shorter hospitalization [4]. However, an estimated 2–6% of stem cell donors are poor mobilizers for G-CSF [5–8]. An insufficient number of HSCs could lead to failure of HSC transplantation and mortality of the recipients [9, 10], suggesting an urgent need to improve the HSC mobilization regimen.

HSCs reside in BM after birth. BM is a complex organ containing different stromal cells to help HSCs with self-renewal, un-differentiation or differentiation and retain in BM [11–13]. Cell adhesion receptors on stromal cells [e.g., N-cadherin, stem cell factor, angiopoietin, stromal-derived factor-1 (SDF-1), vascular cell adhesion molecules-1, intercellular adhesion molecules-1–3, thrombopoietin, osteopontin, E-selectin, and P-selectin] form retention contacts with their counterparts [e.g., N-cadherin, c-kit, tie2, CXC chemokine receptor (CXCR4), very late antigen-4, leukocyte function antigen 1, thrombopoietin receptor, α and β integrin, E-selectin ligand-1, and P-selectin glycoprotein ligand-1 (PSGL-1)] expressed on the surfaces of HSCs to maintain these cells in BM [4, 12]. In response to tissue/organ damage or inflammation, HSCs mobilize to the peripheral blood and reside on the damaged tissue/organ [14, 15]. For clinical use, HSCs can be isolated directly from BM or mobilized to the peripheral blood by inducers, such as G-CSF.

G-CSF regulates granulopoiesis and prevents neutropenia in patients after chemotherapy; it is now the clinical routinely used inducer to mobilize HSCs for transplantation [16–18]. However, the detailed mechanism of how G-CSF induces HSC mobilization remains elusive. Reports suggested five non-mutually exclusive but not completely unified mechanisms involved in G-CSF-induced HSC mobilization [19]. 1. G-CSF activates neutrophil proteases (e.g., matrix metalloproteinases, neutrophil elastase, and cathepsin G) to cleave and inactivate the retention axes [20–24]. 2. G-CSF reduces the expression of SDF-1 on stromal cells by activating the sympathetic neurons or macrophages in BM, resulting in HSC mobilization [25]. 3. G-CSF activates innate immune cells, activating the inflammasome and releasing interleukin (IL)-1β, IL-18, and danger-associated molecular patterns. These molecules then activate the complement system through the mannan-binding lectin-dependent pathway. Subsequently, sphingosine-1-phosphate (S1P) is released from complement-lysed erythrocytes, serving as a chemoattractant for HSC mobilization [26–29]. 4. G-CSF increases the expression and activity of dipeptidyl peptidase 4 (DPP4/CD26) on the surface of endothelial cells. CD26 truncates the neurotransmitter neuropeptide Y (NPY), and the truncated NPY reduces the expression of VE-cadherin and CD31, resulting in HSC trans-endothelial migration [30, 31]. 5. Recently, a paper suggested that nociceptive nerves are required for G-CSF-induced HSC mobilization [32]. AMD3100 (Plerixafor) is another clinically used inducer to mobilize HSCs. As a CXCR4 antagonist, it can bind to CXCR4 and interfere with one of the retention axes (SDF-1 and CXCR4) [33]. Treatments of AMD3100 result in more rapid mobilization of HSCs than that of G-CSF. However, the efficiency of HSC mobilization by AMD3100 alone is much less than that of G-CSF. Therefore, AMD3100 serves as an accessory treatment in combination with G-CSF to induce a more effective mobilization for clinical use, especially in heavily G-CSF-pretreated patients with lymphoma and myeloma [33–35].

P-selectin and PSGL-1 are another retention axes to maintain HSCs in BM [12, 36]. P-selectin is expressed in platelets and endothelial cells [37, 38]. Soluble P-selectin (sP-sel) can be released into circulation by alternative splicing form lacking the trans-membrane domain or proteolytic cleavage of the ectodomain of P-selectin by matrix metalloproteinase [39–41]. Increased plasma sP-sel can be found in patients with hematological malignancies after G-CSF treatments [42, 43]. In addition, G-CSF induced myeloid cell mobilization in PSGL-1 deficiency mice [44] and reduced PSGL-1 expression on the surfaces of human neutrophils [45]. This evidence suggests that P-selectin plays a critical role in G-CSF-induced HSC mobilization.

In this study, the plasma levels of sP-sel in humans and mice before and after G-CSF administration were analyzed. In the mouse model, the role of P-selectin in G-CSF-induced HSC mobilization was analyzed using the loss-of-function (G-CSF treatments in *Selp*^−/−^ mice) and gain-of-function approaches (injection of sP-sel in wild-type mice).

## Methods

### Human sample collection

For stem cell mobilization, G-CSF (Filgrastim, Kirin Brewery Co., Tokyo, Japan) was given subcutaneously daily (10 μg/kg) for 5 consecutive days. Residual specimens from clinical peripheral blood examination of stem cell donors after the last dose of G-CSF administration were collected. Peripheral blood (10 mL) was provided by stem cell donors 1–2 months before G-CSF administrations. Peripheral blood was collected via venipuncture using anticoagulant K_2_EDTA-coated purple-top tubes. Peripheral blood (1–5 mL) was twofold diluted with phosphate-buffered saline (PBS) and layered on 5 ml Ficoll–Paque PLUS (GE Healthcare). Plasma fractions were collected after centrifugation (400 × g for 40 min) at room temperature and used for sP-sel detection.

### Animals

C57BL/6J mice were purchased from the National Laboratory Animal Center (Taipei, Taiwan). P-selectin deficiency mice (*Selp*^−/−^; B6.129S-*Selp*^*tm1Bay*^/J) were obtained from Jackson Laboratory (Bar Harbor, ME) [46]. All animals were maintained in a specific pathogen-free environment in the experimental animal center of Tzu Chi University (Hualien, Taiwan). The experimental protocols involving mice followed the ARRIVE guidelines (Animal Research: Reporting of In Vivo Experiments). These protocols were approved by the Institutional Animal Care and Use Committee of Tzu Chi University under approval IDs 104105, 107085-B, and 109073. Before performing retro-orbital injections or withdrawals, a drop of an anesthetic agent, specifically proparacaine hydrochloride (0.5% Alcaine), was gently applied to the mice's eyeballs. This step was taken to minimize discomfort and pain during the procedure [47]. After the experiments were concluded, the mice were placed in a euthanasia chamber (five mice per 8.5 L) and euthanized using 100% carbon dioxide at a flow rate of 20–30% of the chamber's volume per minute. This gradual flow rate ensured the loss of consciousness without causing pain [48]. Once the animals had lost consciousness, the flow rate could be increased to 40% of the chamber's volume per minute if necessary. The entire process was completed within 5–6 min and adhered to the guidelines established by the Institutional Animal Care and Use Committee of Tzu Chi University.

### G-CSF and P-selectin injections in mice

C57BL/6J mice (male, 11–20 weeks of age) and *Selp*^−/−^ mice (male, 10–19 weeks of age) were subcutaneously injected with 250 μg/kg/day of G-CSF (Filgrastim, in 200 μL normal saline) or the same volume of normal saline for 4 consecutive days. C57BL/6J mice (male, 12–15 weeks of age) were retro-orbitally injected with 0.1, 0.5, or 1 mg/kg of sP-sel [purified mouse P-selectin-IgG fusion protein (BD Pharmingen) or recombinant mouse P-selectin/CD62P Fc chimera (R & D systems) used in Fig. 5, in 200 μL saline] two times a day for 1 day. According to the manufacturer’s instructions, the P-selectin-IgG fusion protein comprises the signal sequence, the lectin domain, the epidermal growth factor-like repeat, and the first two complement-binding domains of mouse P-selectin (CD62P) fused to the Fc region (hinge, CH2, and CH3) of human IgG1 (P-selectin-IgG-Fc). The Fc fusion protein was designed to increase the half-life in the circulation without reducing biological efficacy [49, 50], and the recombinant protein was purified from tissue culture supernatant or ascites by affinity chromatography. As antibodies (IgGs) can bind to specific blood and immune cells expressing receptors for the Fc portion of antibodies (FcRs) [51, 52], the potential involvement of Fc-Fc receptor binding in soluble P-selectin (P-selectin-IgG-Fc)-mediated HSC mobilization was investigated. Human IgG Fc fragment (Jackson ImmunoResearch Laboratories) was injected into mice as a control group at 1 mg/kg two times a day for 1 day. Peripheral blood was retro-orbitally obtained 1 week before injection, on day 2 after the second G-CSF injection, or 17 h after the last G-CSF or sP-sel and IgG Fc injection. Peripheral blood (200 μL) and anticoagulant acid citrate dextrose formula A (ACD-A, 300 μL) were mixed well and loaded on top of 5 mL Ficoll–Paque PLUS. Plasma fraction and mononuclear cell fraction were collected after centrifugation (400 × g for 40 min) at room temperature and used for sP-sel detection and LSK cell analysis, respectively.

### Detection of soluble P-selectin in humans and mice

Human and mouse plasma were twofold and 40-fold diluted with PBS, respectively. The levels of human and mouse sP-sel were measured using a custom-made ProcartaPlex Immunoassay (Thermo Fisher Scientific, Waltham, MA, USA) and enzyme-linked immunosorbent assay (ELISA) in accordance with the manufacturer’s instructions (mouse P-selectin ELISA kit, RayBiotech, Inc., USA), respectively.

### Detection of P-selectin in microparticle and soluble fraction

*Selp*^−/−^ mice (male, 10–19 weeks of age) were retro-orbitally injected with platelets from wild-type C57BL/6J mice at 1 × 10^8^ per mouse per day. This injection was administered for 3 consecutive days in conjunction with the last three doses of G-CSF (250 μg/kg/day). The platelet purification and transfer procedures followed the prior study’s methods [53]. After Ficoll–Paque PLUS purification, the mouse plasma underwent two rounds of centrifugation at room temperature, each at 9170 × g for 10 min, to eliminate any residual platelets. Subsequently, the supernatant underwent ultra-centrifugation at 157,000 × g for 1.5 h at 4 °C to segregate microparticles (in the pellet) from the microparticle-free supernatant [54]. The concentrations of P-selectin in microparticle and soluble fraction were determined using the mouse P-selectin ELISA kit (RayBiotech, Inc. USA).

### Analysis of LSK cells in peripheral blood

Mononuclear cells isolated from the peripheral blood were counted and incubated with 5% bovine serum albumin (Sigma–Aldrich) at 37 °C for 1 h to block nonspecific binding. Then, they were incubated with antibodies against mouse lineage conjugated with fluorescein isothiocyanate (FITC) (BioLegend), mouse Ly-6A/E (stem cell antigen-1, Sca-1) conjugated allophycocyanin (APC) (BioLegend), and mouse CD117 (c-Kit) conjugated with phycoerythrin/Cy7 (PE/Cy7) (BioLegend) or mouse P-selectin conjugated with PE (eBioscience) at 4 °C for 30 min. After the cells were washed with PBS, they were resuspended in 300 μL PBS and analyzed using a flow cytometer (Gallios, Beckman Coulter Life Sciences).

### Detection of white blood cell (WBC) in mice

Mouse peripheral blood (200 μL) and anticoagulant ACD-A (300 μL) were mixed well, and then 25 μL was taken and mixed with 75 μL ACD-A. WBC count was measured using an automated hematology analyzer (XP-300, Sysmex Corporation).

### Statistics

Data are presented as median (Q1 and Q3) or mean ± standard deviation (SD) depending on their distribution (normal or not). Statistical analyses were conducted on Microsoft Office Excel 2003. Comparisons between groups were conducted using two-tailed Student’s *t*-test. A *P* value of less than 0.05 was considered statistically significant. Graphs of human samples were created on GraphPad Prism 5 software. The significance of human samples was analyzed by paired t-test or Mann–Whitney U test depending on its distribution (normal or not). G*Power version 3.1.9.2 was used to calculate the mouse sample size needed [55, 56]. For example, a one-sided test was performed to test the hypothesis that wild-type platelets could ameliorate HSC mobilization defect in *Selp*^−/−^ mice, with an effect size of 1.66, an α of 0.05, and a power (1-β) of 0.80. The estimated sample size was 12 (i.e., 6 per group). One-sided test was also conducted to examine the hypothesis that the LSK cell counts after 0.1 mg/kg sP-sel injection are less than those after G-CSF treatment. An effect size of 2.97, an α of 0.05, and a power (1-β) of 0.80, and a sample size ratio of 1 were set, and the estimated sample size was 6 (i.e., 3 per group).

## Results

### Soluble P-selectin levels were positively associated with HSC mobilization and upregulated in plasma after G-CSF treatments

**Fig. 1:**
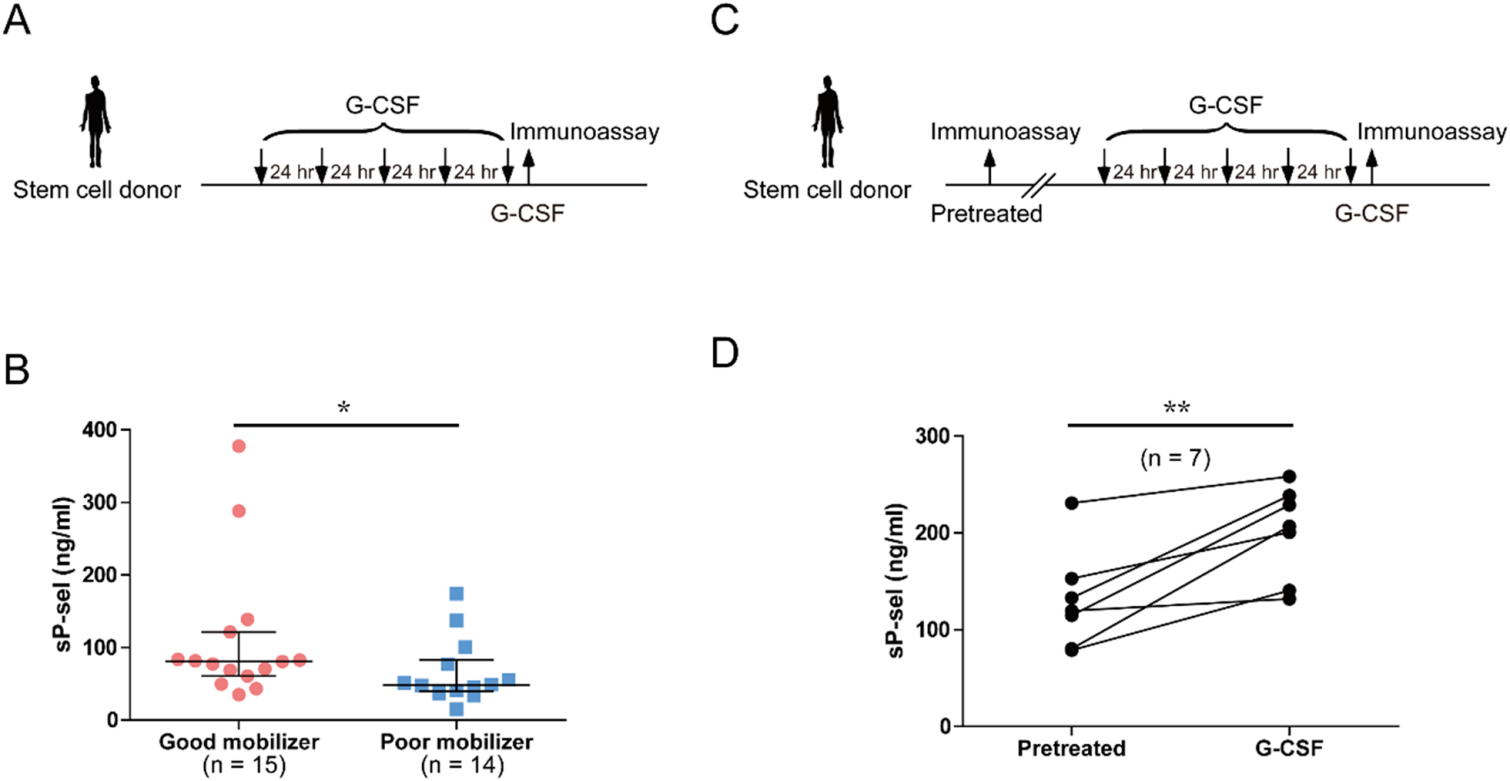
Correlation between plasma soluble P-selectin and hematopoietic stem cell mobilization. The experimental outline for measuring plasma sP-sel in stem-cell donors post-G-CSF administration is shown (**A**). Using ProcartaPlex Immunoassay, sP-sel levels were detected in 15 good and 14 poor mobilizers after 5 consecutive days of G-CSF treatments (**B**). Each closed circle and closed square represents an individual with good or poor mobilization. The data are presented as median (Q1 and Q3). The experimental outline for measuring plasma sP-sel in stem cell donors before and after G-CSF administrations is shown (**C**). Using ELISA, the levels of sP-sel were detected in 7 paired donors before and after 5 consecutive days of G-CSF administration (**D**). The data from the same donor are line-paired. *P < 0.05 and **P < 0.01 compared with the indicated groups.

From August 2015 to January 2018, a total of 309 HSC donors were recruited and categorized as good mobilizers (circulating CD34^+^ cells in peripheral blood ≥ 180/μL, *n* = 15, 4.85%) and poor mobilizers (CD34^+^ cells ≤ 25/μL, *n* = 14, 4.53%) in accordance with the number of CD34^+^ cells in their peripheral blood after five doses of G-CSF administration [57]. Residual specimens from the routine clinical examinations of the donors after the last dose of G-CSF treatments in Hualien Tzu Chi Hospital were collected. Twenty-six soluble proteins related to adhesion molecule (including sP-sel) and inflammatory factors were examined using a custom-made ProcartaPlex Immunoassay (Fig. 1A, experiment outline for post-G-CSF administration) to check whether the levels of soluble factors in plasma are correlated with HSC mobilization. The results revealed that good mobilizers have higher sP-sel in plasma than poor mobilizers (Fig. 1B). Although previous studies suggested that gender and age are associated with HSC mobilization [5, 7, 58–61], the difference remained after adjustment for age and gender by multiple linear regression (Additional file 1: Table S1). Paired samples (before and after G-CSF administration) were obtained from the same donors to examine whether sP-sel is induced by G-CSF. Collecting the paired samples was not easy compared with collecting post-G-CSF samples. Therefore, the paired samples were much less than the post-G-CSF samples (*n* = 7). The sP-sel levels of the paired plasma samples from the same cohort of stem cell donors before (pretreated) and after G-CSF administration (G-CSF) were analyzed using ELISA (Fig. 1C, experiment outline for the paired samples). Pretreated samples were isolated from stem cell donors 1–2 months before G-CSF administration. The data revealed that compared with those in the pretreated stage, the circulating sP-sel levels were markedly upregulated in the plasma of HSC donors after G-CSF administration (Fig. 1D). The levels of sP-sel were positively associated with HSC mobilization and upregulated in plasma after G-CSF administration in healthy stem-cell donors.

### Soluble P-selectin levels were upregulated after G-CSF treatments in mice

**Fig. 2:**
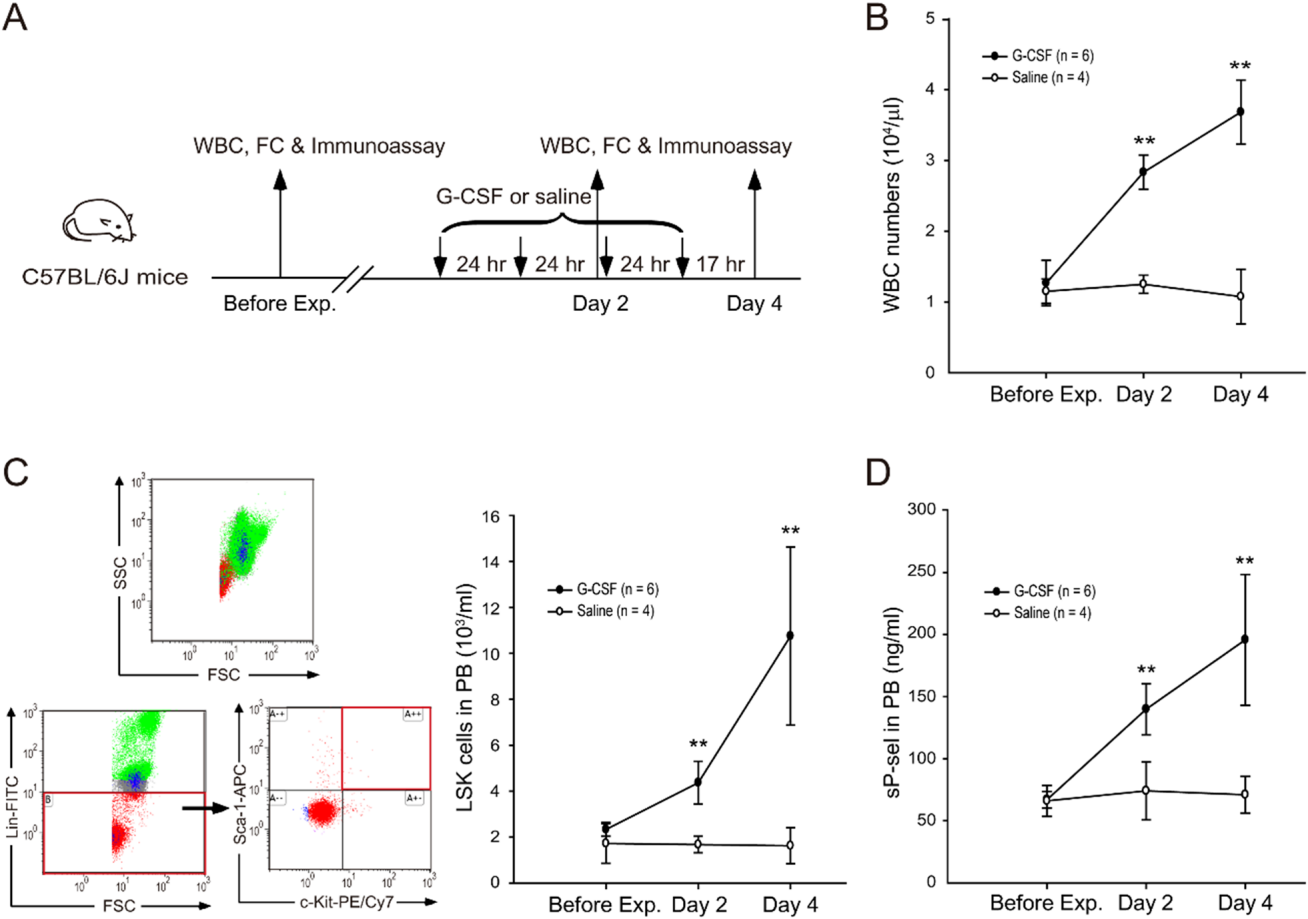
Levels of white blood cell numbers, LSK cells, and plasma soluble P-selectin in G-CSF-treated mice. The experimental outline is shown (**A**). Peripheral blood was collected before and on day 2 and 4 after G-CSF treatments (250 μg/kg/day, *n* = 6, two experiments with six mice per group). Normal saline-treated mice (*n* = 4, two experiments with four mice per group) served as negative controls. White blood cell (WBC) numbers (**B**), LSK (Lin^*−*^/Sca-1^+^/c-Kit^+^) cell numbers (**C**), and plasma sP-sel (**D**) were detected using hematology analysis, flow cytometry (FC), and ELISA, respectively. Data are reported as mean ± standard deviation (SD). **P < 0.01 compared with the before experiment groups (Before Exp)

Due to time consumption in collecting sufficient clinical paired donors, the convenience of collecting samples during G-CSF administrations, and the possibility of using gene-deficiency mice to confirm the importance of P-selectin in HSC mobilization, a mouse model for G-CSF-induced HSC mobilization was established on the basis of previous studies [62–66]. In the present study, male mice were used to rule out the influence of gender in HSC mobilization. C57BL/6 J mice were subcutaneously injected with 250 μg/kg G-CSF daily for 4 consecutive days. Peripheral blood was taken 1 week before the first G-CSF injection, on day 2 (after the second G-CSF injection), and on day 4 (after the last G-CSF injection). Normal saline-treated mice served as negative vehicle controls (Fig. 2A). After the treatments, the number of mouse WBC markedly increased on days 2 and 4 (Fig. 2B). Hematopoietic Lin^−^Sca-1^+^c-Kit^+^ (LSK) cells, which express no lineage-specific markers (e.g., lymphocytes, monocytes/macrophages, granulocytes, natural killer cells, and erythrocytes)-lineage negative (Lin^−^), but have Sca-1 and c-kit (stem cell factor receptor or CD117) expressed on their surfaces, are equivalent to human CD34^+^ HSCs [67, 68]. They were found to be considerably increased in the circulation after G-CSF administrations (Fig. 2C). By contrast, the normal-saline-treated mice showed no obvious increase in WBC and LSK levels in the circulation (Figs. 2B and 2C). In agreement with the human study (Fig. 1B), the established animal model showed upregulated plasma sP-sel levels after G-CSF treatments, similar to humans (Fig. 2D), compared with the normal-saline-treated mice.

### G-CSF-induced HSC mobilization was defective in P-selectin deficiency mice

**Fig. 3:**
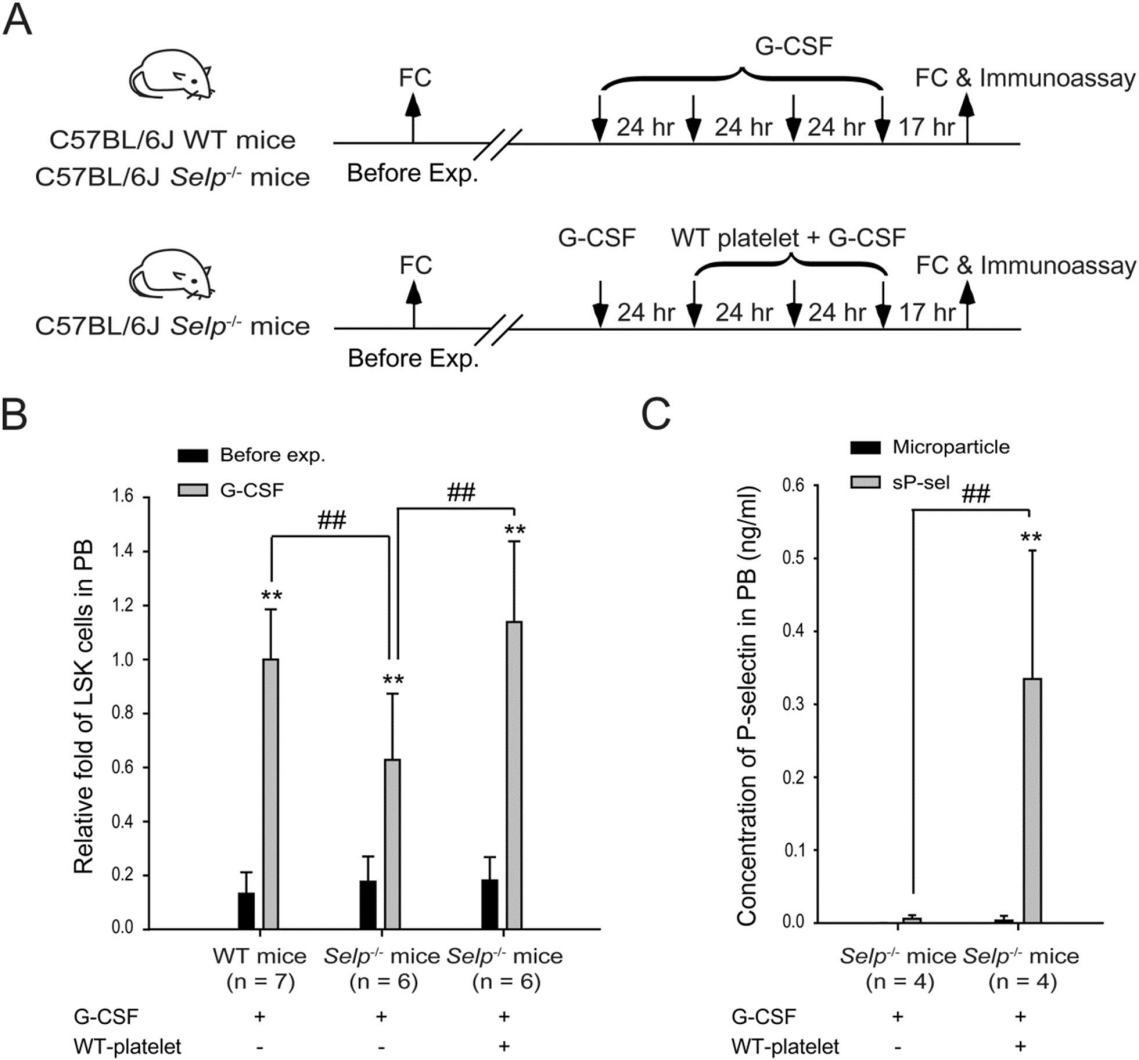
Comparison of LSK cells and P-selectin in peripheral blood between C57BL/6J wild-type and P-selectin-deficiency mice. The experimental outline used to monitor LSK cells and P-selectin in peripheral blood is illustrated (**A**). C57BL/6J wild-type (WT) mice (*n* = 7, five experiments with seven mice per group) and P-selectin-deficiency mice (*Selp*^−/−^; *n* = 6, five experiments with six mice per group) were injected with G-CSF (250 μg/kg/day) for 4 consecutive days. P-selectin-deficiency mice (*Selp*^*−*/*−*^; *n* = 6, five experiments with six mice per group) were injected with WT platelets (1 × 10^8^) once a day, together with the last three doses of G-CSF. LSK cell numbers were detected using flow cytometry (FC) 1 week before and on day 4 after G-CSF treatment. The LSK cell numbers of G-CSF treated-WT mice were set as onefold. Each group’s relative folds of LSK cell numbers are shown (**B**). The concentrations of P-selectin in either microparticles or soluble forms in the peripheral blood of each group were further characterized using immunoassays (**C**). Two groups were included in the study: *Selp*^*−*/*−*^ mice injected with G-CSF (*n* = 4, two experiments with four mice per group) and *Selp*^*−*/*−*^ mice injected with G-CSF and WT platelets (*n* = 4, two experiments with four mice per group). Data are reported as mean ± SD. **P < 0.01 compared with the microparticle groups within each group. ^##^*P* < 0.01 compared with the indicated groups.

Wild-type and P-selectin deficiency (*Selp*^−/−^) mice were used to characterize whether P-selectin is required for G-CSF-induced HSC mobilization (Fig. 3A). The data indicated that G-CSF can mobilize LSK cells to the peripheral blood in *Selp*^−/−^ mice. However, the LSK cell number was markedly lower than that in wild-type mice (about 0.68-fold, Fig. 3B). These results suggested that P-selectin is critical for G-CSF-induced HSC mobilization, and sP-sel may be one of the G-CSF-downstream native inducers for conducting HSC mobilization. P-selectin is almost exclusively expressed and stored in the α-granule of platelet and the Weibel–Palade body of endothelial cells [41]. Soluble P-selectin can be released into circulation by alternative splicing forms lacking the trans-membrane domain or proteolytic cleavage of the ectodomain [41]. *Selp*^−/−^ mice were injected with G-CSF together with platelets collected from wild-type mice to check whether G-CSF-induced sP-sel is derived from platelet (Fig. 3A). The result indicated that wild-type platelets can ameliorate HSC mobilization defect in *Selp*^−/−^ mice (Fig. 3B). High-speed centrifugation was applied to remove residual platelets from the plasma to study the influence of G-CSF on sP-sel release from platelets. The plasma was then separated into microparticle and soluble fractions. The data revealed that in the presence of G-CSF administration, the wild-type platelet-transferred-*Selp*^−/−^ mice exhibited significantly more sP-sel than the G-CSF-treated *Selp*^−/−^ mice (Fig. 3C). These findings underscored the necessity of G-CSF-induced platelet-derived sP-sel for HSC mobilization.

### Soluble P-selectin alone could induce HSC mobilization

**Fig. 4:**
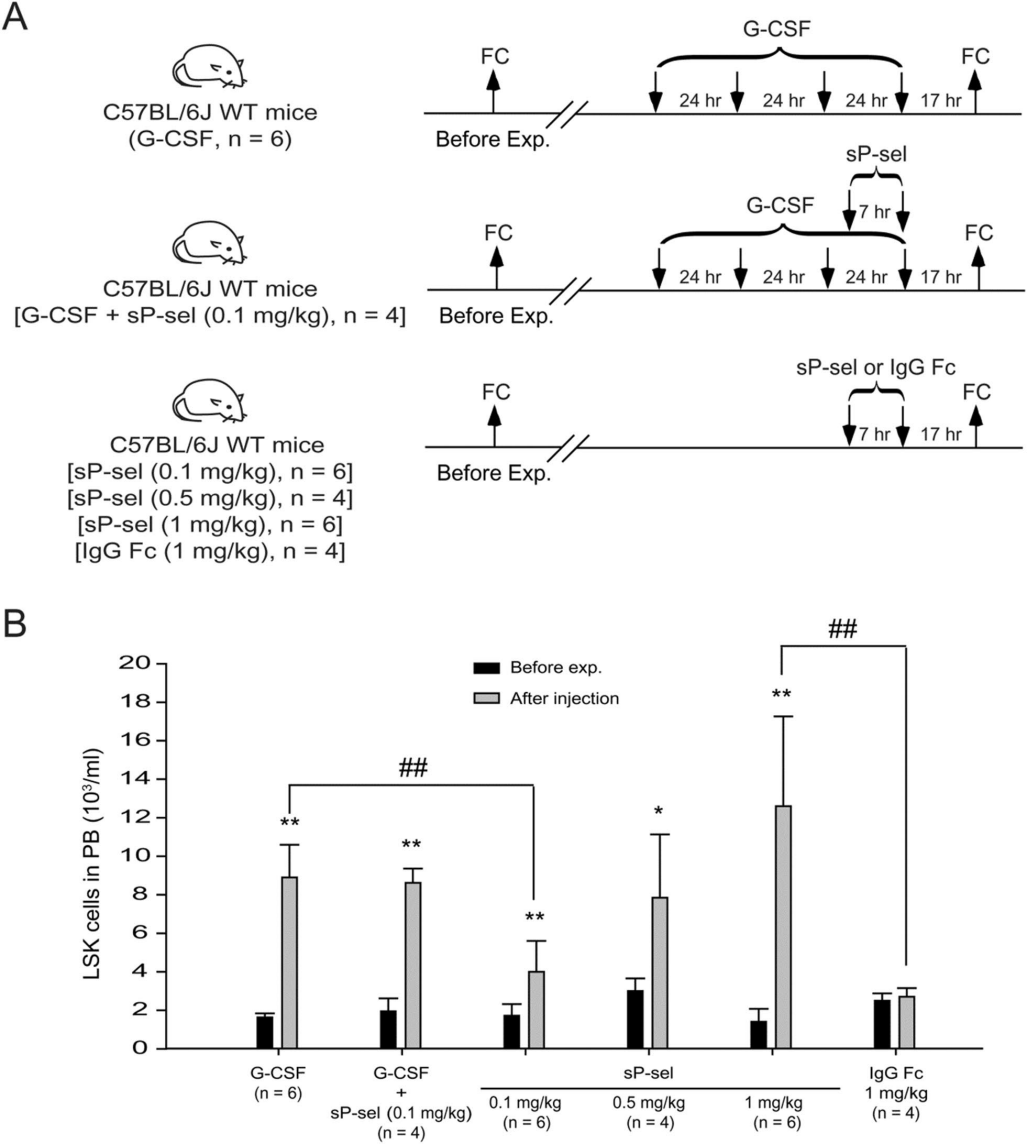
Comparison of LSK cell numbers in the peripheral blood between mice treated with G-CSF, soluble P-selectin, and combined G-CSF and soluble P-selectin. The experimental outline for monitoring LSK cells in the peripheral blood is illustrated (**A**). C57BL/6J wild-type (WT) mice were injected with 4 consecutive days of G-CSF (250 μg/kg/day, *n* = 6, three experiments with six mice per group), combined G-CSF (250 μg/kg/day) and sP-sel (0.1 mg/kg, *n* = 4, one experiment with four mice per group), or three different doses of sP-sel (0.1 mg/kg, *n* = 6, two experiments with six mice per group; 0.5 mg/kg, *n* = 4, two experiments with four mice per group; 1.0 mg/kg, *n* = 6, two experiments with six mice per group). C57BL/6J mice injected with human IgG Fc fragment (1 mg/kg, *n* = 4, one experiment with four mice per group) served as a control group. The LSK cells in peripheral blood were analyzed and quantified using flow cytometry (FC) (**B**). Data are reported as mean ± SD. *P < 0.05 and **P < 0.01 compared with the before experiment groups (Before Exp.). ^##^*P* < 0.01 compared with the indicated groups.

All the above data indicated that sP-sel is one of the downstream mediators of G-CSF. Next, whether sP-sel alone is sufficient for inducing HSC mobilization in mice and whether G-CSF and sP-sel synergistically affect HSC mobilization were investigated. Wild-type mice were subcutaneously injected with G-CSF (250 μg/kg/day) for 4 consecutive days. Additionally, they were treated with recombinant sP-sel (0.1, 0.5, or 1 mg/kg) two times daily for 1 day. Another group of mice received combined treatment of G-CSF and sP-sel (0.1 mg/kg, Fig. 4A). Although the LSK cell counts in the group injected with 0.1 mg/kg of sP-sel were lower than those in the G-CSF treatment group, a significant increase in the number of LSK cells was observed in all groups following treatment (Fig. 4B). The critical finding is that compared with four doses of 4-day treatments of G-CSF, the other two doses (0.5 and 1 mg/kg) of 1-day treatments of sP-sel alone can sufficiently induce comparable LSK cell mobilization (Fig. 4B). The combined treatment of G-CSF and sP-sel resulted in LSK cell counts that were comparable to those observed in G-CSF treatment alone (Fig. 4B). This finding suggested that G-CSF and sP-sel do not have a synergistic effect on HSC mobilization. Due to the generation of sP-sel through the fusion of human IgG Fc portion to enhance its half-life in circulation, precautions were implemented to eliminate any potential immunological reactions caused by the Fc portion of IgG1 binding to Fc receptors on immune cells [51, 52]. As part of the negative control, the human IgG Fc portion was injected two times a day for one day (Fig. 4A). The data revealed that the IgG Fc fragment was unable to induce HSC mobilization (Fig. 4B). These findings suggested that the observed HSC mobilization is specifically attributed to soluble P-selectin rather than the IgG Fc component.

### Soluble P-selectin mobilized HSCs through disrupting the interaction between HSCs and stromal cells in BM

**Fig. 5:**
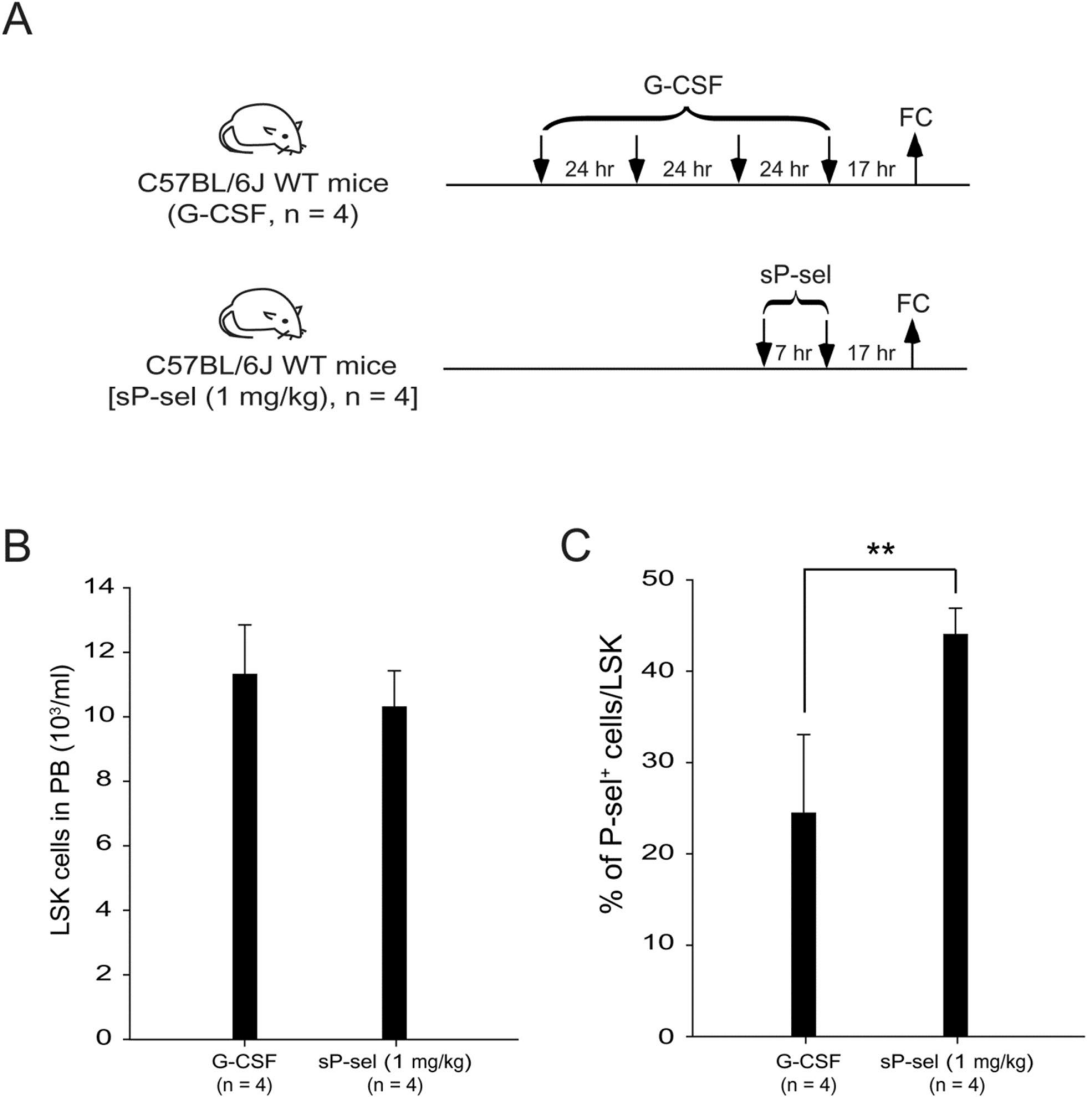
Comparison of P-selectin positive cell percentages in LSK populations between G-CSF- and soluble-P-selectin-treated groups. The experimental procedure for assessing the percentage of P-selectin positive (P-sel^+^) cells in LSK groups is shown (**A**). C57BL/6J wild-type (WT) mice were injected with either 4 consecutive days of G-CSF (250 μg/kg/day, *n* = 4, one experiments with four mice per group) or two doses of sP-sel (1 mg/kg, *n* = 4, one experiments with four mice per group). The LSK cell counts in peripheral blood (**B**) and the percentages of P-sel^+^ cells within LSK populations (**C**) were determined and quantified using flow cytometry (FC) 17 h after the final injection. Data are presented as mean ± SD. ^**^P < 0.01 compared with the indicated groups

P-selectin and E-selectin on endothelial cells and P-selectin on megakaryocytes in BM interact with PSGL-1 on HSCs, crucially contributing to HSC retention [69–73]. This study hypothesized that sP-sel binds to PSGL-1 on HSCs, disrupting the interactions and facilitating the mobilization of HSCs into the peripheral blood. An experiment was designed to test this hypothesis (Fig. 5A). The results demonstrated that in the G-CSF- and sP-sel-treated groups, a comparable mobilization of LSK cells into the peripheral blood was observed (Fig. 5B). However, the percentages of P-sel^+^ cells within the LSK populations significantly increased in the sP-sel administration group (Fig. 5C). This finding strongly supported the notion that sP-sel promotes HSC mobilization by binding to LSK cells and disrupting the interactions between HSCs and stromal cells within BM.

## Discussion

Clinical studies showed that circulating sP-sel levels were upregulated after G-CSF administration in pathological conditions, such as hematological malignancies [42, 43]. However, whether the sP-sel levels also increase after G-CSF administration in healthy stem-cell donors remains uncertain. The present study demonstrated that G-CSF administration induced higher plasma sP-sel levels in healthy humans and a mouse HSC-mobilization model. Moreover, the levels of sP-sel were positively associated with HSC mobilization. In addition, P-selectin deficiency negatively affected G-CSF induced-HSC mobilization, and G-CSF-induced wild-type platelet-derived sP-sel can ameliorate the HSC mobilization defect caused by P-selectin deficiency. Notably, compared with the four administrations of G-CSF (4-day period), only two injections of sP-sel (1-day period) showed similar LSK cell counts in the peripheral blood, suggesting the superior effect of sP-sel over G-CSF on HSC mobilization. In addition, the experiments provided evidence that sP-sel facilitates HSC mobilization by disrupting the crucial interactions between HSCs and stromal cells in BM.

In clinical settings, G-CSF is a routinely used agent for HSC mobilization. However, 5 continuous days are required to proceed with the whole injection process due to the low efficiency and short half-life of G-CSF (about 3–4 h) [74, 75]. The ideal mobilization regimen should be fast, efficient, and natural with fewer adverse effects and not needing multiple injections over several days. The mouse model in this study demonstrated that a more convenient 1-day injection course may improve such 4-day processes of G-CSF administration through sP-sel administration. Fast HSC mobilization was also demonstrated in recent reports. For example, a single injection of CXC chemokine receptor 2 agonist-growth-regulated protein β and AMD3100 and a single oral dose of Viagra combined with a single injection of AMD3100 resulted in rapid HSC mobilization within 15 min and 2 h, respectively [76, 77]. Although the above two regimens can mobilize HSCs rapidly within a short time course, growth-regulated protein β or Viagra alone is unable to achieve the effect unless combined with AMD3100. However, some recipients who received AMD3100-mobilized stem cells have secondary myelodysplastic syndrome/acute myeloid leukemia symptoms [78, 79]. As a native host factor, sP-sel is highly compatible with the human body. Reports showed upregulated sP-sel levels in the circulation of patients with inflammatory conditions, such as hypertension [39], cardiovascular disease [40], rheumatic mitral stenosis [80], myocardial infarction [81], and sepsis [82]. Consistently, animal experiments suggested that the sP-sel levels were upregulated in mice with inflammatory burdens, such as under anthrax lethal toxin [46] and snake venom treatments [83]. These studies suggested that sP-sel can be a native factor for conducting anti-inflammatory and repairing effects. In the present study, sP-sel (two injections only in 1 day) can be a native inducer to mobilize HSCs within one day, comparable with G-CSF administration, which typically takes 4 days. Notably, the potential side effects of high sP-sel concentration in the human body should be a concern when the regimen is applied to clinical medicine in the future.

Inhibition of P-selectin and PSGL-1 interaction is considered a feasible strategy to manage severe inflammation, because the interaction of P-selectin/PSGL-1 is a critical step to initiate the binding of circulating leukocytes to the endothelium and infiltrate into the inflamed tissues [84–89]. Similarly, it has been postulated that the P-selectin-PSGL-1 interaction between HSCs and BM stroma cells is necessary for HSCs retention in BM [12]. Accordingly, in the present study, sP-sel served as a native competitor and antagonist for disrupting HSC-stromal cell interaction, thus facilitating HSC mobilization. The presence of HSCs in the BM of *Selp*^−/−^ mice suggested that other HSC-stromal cell interaction machinery supports BM residential HSCs. However, given that the P-selectin defect reduced the G-CSF-mediated HSC mobilization, the P-selectin pathway may play a crucial role in regulating HSC stability and motility in BM. P-selectin is primarily expressed and stored in the α-granules of platelets and the Weibel–Palade bodies of endothelial cells [41]. P-selectin on the surface of endothelial cells in BM also plays a vital role in hematopoietic progenitor cells residing in BM [71, 72]. The endothelial cells in BM serve as a physical barrier for HSC trafficking and as stromal cells to retain HSCs in BM [30, 31, 90]. Loss of the endothelial transmembrane protein Robo4 or activating vascular endothelial growth factor receptor 2 facilitates HSC mobilization from BM to peripheral blood [91, 92], suggesting that the endothelial cells in BM may play a crucial role in HSC mobilization. A notable detail that the P-selectin-deficiency mice were not specifically engineered to have a tissue-specific knockout, so P-selectin deficiency was observed across all P-selectin-expressing cell types, including platelets and endothelial cells in mice. However, the findings demonstrated that the defect in HSC mobilization observed in *Selp*^−/−^ mice can be fully restored by transferring wild-type platelets. Furthermore, G-CSF stimulates the release of sP-sel from platelets. Considering that a total of 3 × 10^8^ wild-type platelets only was transfused into the P-selectin-deficiency mice (with approximately 1 × 10^9^/mL platelets), the quantity of sP-sel released by these platelets may be less than that induced by G-CSF treatment in wild-type mice. However, this soluble form of P-selectin, even when secreted in small amounts by platelets, holds the potential to alleviate the HSC mobilization deficiency observed in mice lacking P-selectin. Investigating whether G-CSF directly or indirectly induces platelets to release sP-sel remains a promising avenue for further research. The data suggested that while endothelial cells may contribute to the process, platelets play a dominant role in G-CSF-induced HSC mobilization.

Although numerous reported mechanisms attempt to explain how G-CSF induces HSC mobilization, they are not mutually exclusive and remain largely ununified thus far. Multiple paired molecules between HSCs and stromal cells play a role in retaining HSCs in BM. In this study, elevated circulating levels of sP-sel disrupted the interaction between PSGL-1 and P-selectin on HSCs and stromal cells, ultimately promoting HSC mobilization. This competitive mechanism closely resembles the action of AMD3100, which binds to CXCR4 expressed on the surface of HSCs and disrupts their interactions with stromal cells that express SDF-1 [33, 93]. Comparison with the five mechanisms (Introduction section) that are known to mobilize HSCs by G-CSF administration showed that the mechanism of sP-sel-mediated HSC mobilization is similar to mechanisms 1 and 2, where cleavage or attenuation of retention axes occurs. In mechanism 3, pro-inflammatory factors such as IL-1β and IL-18, are involved in HSC mobilization [26]. The authors’ previous study found that HSC mobilization following G-CSF administration was linked to pro-inflammatory factors such as IL-22, tumor necrosis factor, and interferon-γ [5]. These results align with earlier research on infectious diseases, toxin treatments, and cardiovascular injuries [46, 94, 95], indicating an association between circulating levels of sP-sel and inflammatory conditions. The previously reported pro-survival and tissue-repair functions of sP-sel [46, 83, 96] could be partly attributed to HSC mobilization, highlighting a natural self-rescue feedback mechanism between sP-sel-mediated tissue repair and inflammation-related tissue damage. Meanwhile, S1P was shown to facilitate HSC mobilization [97]. S1P is a pro-inflammatory factor that promotes inflammation by binding to its receptors on immune cells and inducing their activation, infiltration, and pro-inflammatory cytokines [98]. Intriguingly, S1P receptor promotes leukocyte rolling by mobilizing endothelial P-selectin [99], suggesting a regulatory role and crosstalk of S1P pathway on the modulation of endothelial P-selectin expression under inflammation. Regardless of any mechanism, HSCs require mechanism 4 to open the gate of BM and mobilize in peripheral blood. The mechanisms on how G-CSF induces upregulation of sP-sel and any potential crosstalk between other mobilization mechanisms are not yet fully understood, and further investigation is warranted to elucidate the detailed mechanism.

## Conclusions

In conclusion, sP-sel was identified as a novel endogenous agent for mobilizing HSCs. sP-sel injections achieved a faster and more convenient regimen for mobilizing HSCs than G-CSF in mice. These findings could offer valuable insights for future endeavors to develop and optimize the mobilization of human HSCs.

### Supplementary Information

**Additional file 1**. **Table S1.** Association of soluble P-selectin with HSC mobilization after adjustment for age and gender (n = 29).

## Data Availability

All data generated and/or analyzed during this study are available from the corresponding authors upon reasonable request.

## Abbreviations

G-CSF: Granulocyte colony-stimulating factor
HSCs: Hematopoietic stem cells
PSGL-1: P-selectin glycoprotein ligand-1
ELISA: Enzyme-linked immunosorbent assay
sP-sel: Soluble P-selectin
BM: Bone marrow
SDF-1: Stromal-derived factor-1
CXCR4: CXC chemokine receptor 4
IL: Interleukin
S1P: Sphingosine-1-phosphate
*Selp*^−^^/^^−^ mice: P-selectin-deficiency mice
DPP4: Dipeptidyl peptidase 4
NPY: Neuropeptide
PBS: Phosphate-buffered saline
ACD-A: Acid citrate dextrose formula A
SD: Standard deviation
WBC: White blood cell
LSK: Lin^−^Sca-1^+^c-Kit^+^

## References

[1] Orkin SH, Zon LI (2008). Hematopoiesis: an evolving paradigm for stem cell biology. Cell.

[2] Henig I, Zuckerman T (2014). Hematopoietic stem cell transplantation-50 years of evolution and future perspectives. Rambam Maimonides Med J.

[3] Thomas ED, Lochte HL, Lu WC (1957). Intravenous infusion of bone marrow in patients receiving radiation and chemotherapy. N Engl J Med.

[4] Chen J, Lazarus HM, Dahi PB (2020). Getting blood out of a stone: Identification and management of patients with poor hematopoietic cell mobilization. Blood Rev..

[5] Wang TF, Liou YS, Chang HH (2022). Correlation of body mass index and proinflammatory cytokine levels with hematopoietic stem cell mobilization. J Clin Med..

[6] Teipel R, Schetelig J, Kramer M (2015). Prediction of hematopoietic stem cell yield after mobilization with granulocyte-colony-stimulating factor in healthy unrelated donors. Transfusion.

[7] Richa E, Papari M, Allen J (2009). Older age but not donor health impairs allogeneic granulocyte colony-stimulating factor (G-CSF) peripheral blood stem cell mobilization. Biol Blood Marrow Transpl.

[8] Ings SJ, Balsa C, Leverett D (2006). Peripheral blood stem cell yield in 400 normal donors mobilised with granulocyte colony-stimulating factor (G-CSF): impact of age, sex, donor weight and type of G-CSF used. Br J Haematol.

[9] Kiss JE, Rybka WB, Winkelstein A (1997). Relationship of CD34+ cell dose to early and late hematopoiesis following autologous peripheral blood stem cell transplantation. Bone Marrow Transp.

[10] Weaver CH, Hazelton B, Birch R (1995). An analysis of engraftment kinetics as a function of the CD34 content of peripheral blood progenitor cell collections in 692 patients after the administration of myeloablative chemotherapy. Blood.

[11] Anthony BA, Link DC (2014). Regulation of hematopoietic stem cells by bone marrow stromal cells. Trends Immunol.

[12] Beatriz SA, Antonio LV, Carlos LL. Stem Cell Transpl. 2012; Chap 11. 152–70.

[13] Schofield R (1978). The relationship between the spleen colony-forming cell and the haemopoietic stem cell. Blood Cells.

[14] Ratajczak MZ, Kim CH, Wojakowski W (2010). Innate immunity as orchestrator of stem cell mobilization. Leukemia.

[15] Reca R, Cramer D, Yan J (2007). A novel role of complement in mobilization: immunodeficient mice are poor granulocyte-colony stimulating factor mobilizers because they lack complement-activating immunoglobulins. Stem Cells.

[16] de Kruijf EFM, Fibbe WE, van Pel M (2020). Cytokine-induced hematopoietic stem and progenitor cell mobilization: unraveling interactions between stem cells and their niche. Ann N Y Acad Sci.

[17] Hubel K, Dale DC, Liles WC (2002). Therapeutic use of cytokines to modulate phagocyte function for the treatment of infectious diseases: current status of granulocyte colony-stimulating factor, granulocyte-macrophage colony-stimulating factor, macrophage colony-stimulating factor, and interferon-gamma. J Infect Dis.

[18] Gabrilove JL, Jakubowski A, Scher H (1988). Effect of granulocyte colony-stimulating factor on neutropenia and associated morbidity due to chemotherapy for transitional-cell carcinoma of the urothelium. N Engl J Med.

[19] Chang HH, Liou YS, Sun DS (2022). Hematopoietic stem cell mobilization. Tzu Chi Med J.

[20] Levesque JP, Winkler IG, Larsen SR (2007). Mobilization of bone marrow-derived progenitors. Handb Exp Pharmacol.

[21] Levesque JP, Hendy J, Winkler IG (2003). Granulocyte colony-stimulating factor induces the release in the bone marrow of proteases that cleave c-KIT receptor (CD117) from the surface of hematopoietic progenitor cells. Exp Hematol.

[22] Levesque JP, Hendy J, Takamatsu Y (2003). Disruption of the CXCR4/CXCL12 chemotactic interaction during hematopoietic stem cell mobilization induced by GCSF or cyclophosphamide. J Clin Invest.

[23] Heissig B, Hattori K, Dias S (2002). Recruitment of stem and progenitor cells from the bone marrow niche requires MMP-9 mediated release of kit-ligand. Cell.

[24] Levesque JP, Takamatsu Y, Nilsson SK (2001). Vascular cell adhesion molecule-1 (CD106) is cleaved by neutrophil proteases in the bone marrow following hematopoietic progenitor cell mobilization by granulocyte colony-stimulating factor. Blood.

[25] Rankin SM (2012). Chemokines and adult bone marrow stem cells. Immunol Lett.

[26] Lenkiewicz AM, Adamiak M, Thapa A (2019). The Nlrp3 inflammasome orchestrates mobilization of bone marrow-residing stem cells into peripheral blood. Stem Cell Rev Rep.

[27] Tay J, Levesque JP, Winkler IG (2017). Cellular players of hematopoietic stem cell mobilization in the bone marrow niche. Int J Hematol.

[28] Adamiak M, Ratajczak MZ (2017). Innate immunity and mobilization of hematopoietic stem cells. Curr Stem Cell Rep.

[29] Ratajczak MZ, Lee H, Wysoczynski M (2010). Novel insight into stem cell mobilization-plasma sphingosine-1-phosphate is a major chemoattractant that directs the egress of hematopoietic stem progenitor cells from the bone marrow and its level in peripheral blood increases during mobilization due to activation of complement cascade/membrane attack complex. Leukemia.

[30] Singh P, Hoggatt J, Kamocka MM (2017). Neuropeptide Y regulates a vascular gateway for hematopoietic stem and progenitor cells. J Clin Invest.

[31] Itkin T, Gomez-Salinero JM, Rafii S (2017). Open the gates: vascular neurocrine signaling mobilizes hematopoietic stem and progenitor cells. J Clin Invest.

[32] Gao X, Zhang D, Xu C (2021). Nociceptive nerves regulate haematopoietic stem cell mobilization. Nature.

[33] Broxmeyer HE, Orschell CM, Clapp DW (2005). Rapid mobilization of murine and human hematopoietic stem and progenitor cells with AMD3100, a CXCR4 antagonist. J Exp Med.

[34] Flomenberg N, Devine SM, Dipersio JF (2005). The use of AMD3100 plus G-CSF for autologous hematopoietic progenitor cell mobilization is superior to G-CSF alone. Blood.

[35] Romon I, Castillo C, Cid J (2022). Use of plerixafor to mobilize haematopoietic progenitor cells in healthy donors. Vox Sang.

[36] Hidalgo A, Weiss LA, Frenette PS (2002). Functional selectin ligands mediating human CD34(+) cell interactions with bone marrow endothelium are enhanced postnatally. J Clin Invest.

[37] Zarbock A, Polanowska-Grabowska RK, Ley K (2007). Platelet-neutrophil-interactions: linking hemostasis and inflammation. Blood Rev.

[38] Easton AS, Dorovini-Zis K (2001). The kinetics, function, and regulation of P-selectin expressed by human brain microvessel endothelial cells in primary culture. Microvasc Res.

[39] Chen AY, Ha JN, Delano FA (2012). Receptor cleavage and P-selectin-dependent reduction of leukocyte adhesion in the spontaneously hypertensive rat. J Leukoc Biol.

[40] Futh R, Dinh W, Nickl W (2009). Soluble P-selectin and matrix metalloproteinase 2 levels are elevated in patients with diastolic dysfunction independent of glucose metabolism disorder or coronary artery disease. Exp Clin Cardiol.

[41] Cummings RD, Chiffoleau E, van Kyook Y, et al. C-Type Lectins. In: th, Varki A, Cummings RD, Esko JD, Stanley P, Hart GW, et al., editors. Essentials of Glycobiology. Cold Spring Harbor (NY) 2022. p. 455–74.

[42] Nomura S, Inami N, Kanazawa S (2004). Elevation of platelet activation markers and chemokines during peripheral blood stem cell harvest with G-CSF. Stem Cells.

[43] Ohsaka A, Saionji K, Igari J (1998). Granulocyte colony-stimulating factor administration increases serum concentrations of soluble selectins. Br J Haematol.

[44] Miszti-Blasius K, Felszeghy S, Kiss C (2014). P-selectin glycoprotein ligand-1 deficiency augments G-CSF induced myeloid cell mobilization. Naunyn Schmiedebergs Arch Pharmacol.

[45] Jilma B, Hergovich N, Homoncik M (2002). Rapid down modulation of P-selectin glycoprotein ligand-1 (PSGL-1, CD162) by G-CSF in humans. Transfusion.

[46] Sun DS, Chang YW, Kau JH (2017). Soluble P-selectin rescues mice from anthrax lethal toxin-induced mortality through PSGL-1 pathway-mediated correction of hemostasis. Virulence.

[47] Hu Youwei J, Chang B (2015). Effect of cooling proparacaine 0.5% eye drops on patient's comfort during instillation. Eye (Lond)..

[48] Niel L, Stewart SA, Weary DM (2008). Effect of flow rate on aversion to gradual-fill carbon dioxide exposure in rats. Appl Anim Behav Sci.

[49] Kaur S, Sehgal A, Wu AC (2021). Stable colony-stimulating factor 1 fusion protein treatment increases hematopoietic stem cell pool and enhances their mobilisation in mice. J Hematol Oncol.

[50] Gow DJ, Sauter KA, Pridans C (2014). Characterisation of a novel Fc conjugate of macrophage colony-stimulating factor. Mol Ther.

[51] Ben Mkaddem S, Benhamou M, Monteiro RC (2019). Understanding Fc receptor involvement in inflammatory diseases: from mechanisms to new therapeutic tools. Front Immunol.

[52] Huang HS, Sun DS, Lien TS (2010). Dendritic cells modulate platelet activity in IVIg-mediated amelioration of ITP in mice. Blood.

[53] Pethaperumal S, Hung SC, Lien TS (2022). P-selectin is a critical factor for platelet-mediated protection on restraint stress-induced gastrointestinal injury in mice. Int J Mol Sci..

[54] Dunlop LC, Skinner MP, Bendall LJ (1992). Characterization of GMP-140 (P-selectin) as a circulating plasma protein. J Exp Med.

[55] Charan J, Kantharia ND (2013). How to calculate sample size in animal studies?. J Pharmacol Pharmacother.

[56] Faul F, Erdfelder E, Lang AG (2007). G*Power 3: a flexible statistical power analysis program for the social, behavioral, and biomedical sciences. Behav Res Methods.

[57] Gambell P, Herbert K, Dickinson M (2012). Peripheral blood CD34+ cell enumeration as a predictor of apheresis yield: an analysis of more than 1,000 collections. Biol Blood Marrow Transplant.

[58] Ozkurt ZN, Batmaz L, Yegin ZA (2017). Factors affecting hematopoietic stem cell mobilization and apheresis in allogeneic donors: the role of iron status. Transfus Apher Sci.

[59] Lenk J, Bornhauser M, Kramer M (2013). Sex and body mass index but not CXCL12 801 G/A polymorphism determine the efficacy of hematopoietic cell mobilization: a study in healthy volunteer donors. Biol Blood Marrow Transpl.

[60] Chen SH, Yang SH, Chu SC (2011). The role of donor characteristics and post-granulocyte colony-stimulating factor white blood cell counts in predicting the adverse events and yields of stem cell mobilization. Int J Hematol.

[61] Wang TF, Wen SH, Chen RL (2008). Factors associated with peripheral blood stem cell yield in volunteer donors mobilized with granulocyte colony-stimulating factors: the impact of donor characteristics and procedural settings. Biol Blood Marrow Transpl.

[62] Luo C, Wang L, Wu G (2021). Comparison of the efficacy of hematopoietic stem cell mobilization regimens: a systematic review and network meta-analysis of preclinical studies. Stem Cell Res Ther.

[63] Forristal CE, Nowlan B, Jacobsen RN (2015). HIF-1alpha is required for hematopoietic stem cell mobilization and 4-prolyl hydroxylase inhibitors enhance mobilization by stabilizing HIF-1alpha. Leukemia.

[64] Saez B, Ferraro F, Yusuf RZ (2014). Inhibiting stromal cell heparan sulfate synthesis improves stem cell mobilization and enables engraftment without cytotoxic conditioning. Blood.

[65] Lucas D, Bruns I, Battista M (2012). Norepinephrine reuptake inhibition promotes mobilization in mice: potential impact to rescue low stem cell yields. Blood.

[66] Kubonishi S, Kikuchi T, Yamaguchi S (2007). Rapid hematopoietic progenitor mobilization by sulfated colominic acid. Biochem Biophys Res Commun.

[67] Farahbakhshian E, Verstegen MM, Visser TP (2014). Angiopoietin-like protein 3 promotes preservation of stemness during ex vivo expansion of murine hematopoietic stem cells. PLoS ONE.

[68] Zachman DK, Leon RP, Das P (2013). Endothelial cells mitigate DNA damage and promote the regeneration of hematopoietic stem cells after radiation injury. Stem Cell Res.

[69] Eto T, Winkler I, Purton LE (2005). Contrasting effects of P-selectin and E-selectin on the differentiation of murine hematopoietic progenitor cells. Exp Hematol.

[70] Levesque JP, Zannettino AC, Pudney M (1999). PSGL-1-mediated adhesion of human hematopoietic progenitors to P-selectin results in suppression of hematopoiesis. Immunity.

[71] Mazo IB, Gutierrez-Ramos JC, Frenette PS (1998). Hematopoietic progenitor cell rolling in bone marrow microvessels: parallel contributions by endothelial selectins and vascular cell adhesion molecule 1. J Exp Med.

[72] Frenette PS, Subbarao S, Mazo IB (1998). Endothelial selectins and vascular cell adhesion molecule-1 promote hematopoietic progenitor homing to bone marrow. Proc Natl Acad Sci U S A.

[73] Schweitzer KM, Drager AM, van der Valk P (1996). Constitutive expression of E-selectin and vascular cell adhesion molecule-1 on endothelial cells of hematopoietic tissues. Am J Pathol.

[74] Xu F, Zhang Y, Miao Z (2019). Efficacy and safety of mecapegfilgrastim for prophylaxis of chemotherapy-induced neutropenia in patients with breast cancer: a randomized, multicenter, active-controlled phase III trial. Ann Transl Med.

[75] Anderlini P, Przepiorka D, Champlin R (1996). Biologic and clinical effects of granulocyte colony-stimulating factor in normal individuals. Blood.

[76] Hoggatt J, Singh P, Tate TA (2018). Rapid mobilization reveals a highly engraftable hematopoietic stem cell. Cell..

[77] Smith-Berdan S, Bercasio A, Rajendiran S (2019). Viagra enables efficient, single-day hematopoietic stem cell mobilization. Stem Cell Reports.

[78] Tanaka H, Kuwabara C, Kayamori K (2018). Therapy-related myelodysplastic syndrome after autologous stem cell transplantation using plerixafor for mobilized stem cells in a patient with multiple myeloma. J Clin Exp Hematop.

[79] Deol A, Abrams J, Masood A (2013). Long-term follow up of patients proceeding to transplant using plerixafor mobilized stem cells and incidence of secondary myelodysplastic syndrome/AML. Bone Marrow Transplant.

[80] Chen MC, Chang HW, Juang SS (2004). Increased plasma levels of soluble P-selectin in rheumatic mitral stenosis. Chest.

[81] Liu WH, Yang CH, Yeh KH (2005). Circulating levels of soluble P-selectin in patients in the early and recent phases of myocardial infarction. Chang Gung Med J.

[82] Zonneveld R, Martinelli R, Shapiro NI (2014). Soluble adhesion molecules as markers for sepsis and the potential pathophysiological discrepancy in neonates, children and adults. Crit Care.

[83] Sun DS, Ho PH, Chang HH (2016). Soluble P-selectin rescues viper venom-induced mortality through anti-inflammatory properties and PSGL-1 pathway-mediated correction of hemostasis. Sci Rep.

[84] Kutlar A, Ataga KI, McMahon L (2012). A potent oral P-selectin blocking agent improves microcirculatory blood flow and a marker of endothelial cell injury in patients with sickle cell disease. Am J Hematol.

[85] Tardif JC, Tanguay JF, Wright SR (2013). Effects of the P-selectin antagonist inclacumab on myocardial damage after percutaneous coronary intervention for non-ST-segment elevation myocardial infarction: results of the SELECT-ACS trial. J Am Coll Cardiol.

[86] Ley K (2003). The role of selectins in inflammation and disease. Trends Mol Med.

[87] Ataga KI, Kutlar A, Kanter J (2017). Crizanlizumab for the prevention of pain crises in sickle cell disease. N Engl J Med.

[88] Barthel SR, Gavino JD, Descheny L (2007). Targeting selectins and selectin ligands in inflammation and cancer. Expert Opin Ther Targets.

[89] Patel MS, Miranda-Nieves D, Chen J (2017). Targeting P-selectin glycoprotein ligand-1/P-selectin interactions as a novel therapy for metabolic syndrome. Transl Res.

[90] Szade A, Szade K, Mahdi M (2021). The role of heme oxygenase-1 in hematopoietic system and its microenvironment. Cell Mol Life Sci.

[91] Smith-Berdan S, Bercasio A, Kramer L (2021). Acute and endothelial-specific Robo4 deletion affect hematopoietic stem cell trafficking independent of VCAM1. PLoS ONE.

[92] Bisht K, Brunck ME, Matsumoto T (2019). HIF prolyl hydroxylase inhibitor FG-4497 enhances mouse hematopoietic stem cell mobilization via VEGFR2/KDR. Blood Adv.

[93] Liles WC, Broxmeyer HE, Rodger E (2003). Mobilization of hematopoietic progenitor cells in healthy volunteers by AMD3100, a CXCR4 antagonist. Blood.

[94] Schrijver IT, Kemperman H, Roest M (2017). Soluble P-selectin as a biomarker for infection and survival in patients with a systemic inflammatory response syndrome on the intensive care unit. Biomark Insights.

[95] Gross PL (2017). Soluble P-selectin is the smoke, not the fire. Blood.

[96] Kapupara K, Wen YT, Tsai RK (2017). Soluble P-selectin promotes retinal ganglion cell survival through activation of Nrf2 signaling after ischemia injury. Cell Death Dis.

[97] Juarez JG, Harun N, Thien M (2012). Sphingosine-1-phosphate facilitates trafficking of hematopoietic stem cells and their mobilization by CXCR4 antagonists in mice. Blood.

[98] Obinata H, Hla T (2019). Sphingosine 1-phosphate and inflammation. Int Immunol.

[99] Nussbaum C, Bannenberg S, Keul P (2015). Sphingosine-1-phosphate receptor 3 promotes leukocyte rolling by mobilizing endothelial P-selectin. Nat Commun.

