# Supplementary material for: Platelet-derived circulating soluble P-selectin is sufficient to induce hematopoietic stem cell mobilization
Source: Stem Cell Res Ther. 2023 Oct 20;14:300. doi: 10.1186/s13287-023-03527-w (PMC10589967; doi:10.1186/s13287-023-03527-w)
Supplement: Supplementary file 1 — Additional file 1. Table S1. Association of soluble P-selectin with HSC mobilization after adjustment for age and gender (n = 29). [file 13287_2023_3527_MOESM1_ESM.docx]

Supplementary Materials

**Platelet-derived circulating soluble P-selectin is sufficient to induce hematopoietic stem cell mobilization**

Tso-Fu Wang^1,2,3,#^, Yu-Shan Liou^4,#^, Shang-Hsien Yang^2,3,5^, Guan-Ling Lin^4,6^, Ya-Wen Chiang^4^, Te-Sheng Lien^4^, Chi-Cheng Li^1,7^, Jen-Hung Wang^8^, Hsin-Hou Chang^4,*^, Der-Shan Sun^4,*^

^1^ Department of Hematology and Oncology, Hualien Tzu Chi Hospital, Buddhist Tzu Chi Medical Foundation, Hualien, Taiwan, Republic of China.

^2^ Department of Medicine, College of Medicine, Tzu Chi University, Hualien, Taiwan, Republic of China.

^3^ Buddhist Tzu Chi Stem Cells Center, Hualien Tzu Chi Hospital, Buddhist Tzu Chi Medical Foundation, Hualien, Taiwan, Republic of China.

^4^ Department of Molecular Biology and Human Genetics, College of Medicine, Tzu Chi University, Hualien, Taiwan, Republic of China.

^5^ Department of Pediatric Hematology and Oncology, Hualien Tzu Chi Hospital, Buddhist Tzu Chi Medical Foundation, Hualien, Taiwan, Republic of China.

^6^ Present address: Integration Center of Traditional Chinese and Modern Medicine, Hualien Tzu Chi Hospital, Buddhist Tzu Chi Medical Foundation, Hualien, Taiwan, Republic of China.

^7^ Center of Stem Cell & Precision Medicine, Hualien Tzu Chi Hospital, Buddhist Tzu Chi Medical Foundation, Hualien, Taiwan, Republic of China.

^8^ Department of Medical Research, Hualien Tzu Chi Hospital, Buddhist Tzu Chi Medical Foundation, Hualien, Taiwan, Republic of China.

#These authors contributed equally to this work.

*Corresponding authors:

Hsin-Hou Chang, Ph.D. and Der-Shan Sun, Ph.D.

Department of Molecular Biology and Human Genetics, Tzu Chi University,

No. 701, Section 3, Zhong-Yang Road, Hualien 97004, Taiwan, Republic of China.

Phone: 886-3-8565301 ext. 2667 (Hsin-Hou Chang), ext. 2681 (Der-Shan Sun)

FAX: 886-3-8561422

Email: [hhchang@mail.tcu.edu.tw](mailto:hhchang@mail.tcu.edu.tw) (Hsin-Hou Chang); [dssun@mail.tcu.edu.tw](mailto:dssun@mail.tcu.edu.tw) (Der-Shan Sun)

Supplementary Table S1. Association of soluble P-selectin with HSC mobilization after adjustment for age and gender (*n* = 29).

|  | **Crude** | | **Adjusted** | |
| --- | --- | --- | --- | --- |
|  | **β (95% CI) *P*-Value** | | **β (95% CI) *P*-Value** | |
| Age | −0.22 (−4.76, 4.32) | 0.920 | 0.05 (−4.47, 4.57) | 0.982 |
| Gender  (M vs. F) | 13.18 (−46.76, 73.12) | 0.655 | -40.20 (−119.64, 39.24) | 0.307 |
| Group  (Good vs. Poor) | 45.99 (−11.36, 103.35) | 0.111 | 77.35 (−0.60, 155.30) | 0.052 |

Data are presented as β (95% CI). * *P*-value < 0.05 was considered statistically significant after test.
